# Dchs1–Fat4 regulation of polarized cell behaviours during skeletal morphogenesis

**DOI:** 10.1038/ncomms11469

**Published:** 2016-05-05

**Authors:** Yaopan Mao, Anna Kuta, Ivan Crespo-Enriquez, Danielle Whiting, Tina Martin, Joanna Mulvaney, Kenneth D. Irvine, Philippa Francis-West

**Affiliations:** 1 Howard Hughes Medical Institute, Waksman Institute and Department of Molecular Biology and Biochemistry, Rutgers The State University of New Jersey, Piscataway, New Jersey 08854, USA; 2 Department of Craniofacial Development and Stem Cell Biology, King's College London, Dental Institute, London SE1 9RT, UK

## Abstract

Skeletal shape varies widely across species as adaptation to specialized modes of feeding and locomotion, but how skeletal shape is established is unknown. An example of extreme diversity in the shape of a skeletal structure can be seen in the sternum, which varies considerably across species. Here we show that the Dchs1–Fat4 planar cell polarity pathway controls cell orientation in the early skeletal condensation to define the shape and relative dimensions of the mouse sternum. These changes fit a model of cell intercalation along differential Dchs1–Fat4 activity that drives a simultaneous narrowing, thickening and elongation of the sternum. Our results identify the regulation of cellular polarity within the early pre-chondrogenic mesenchyme, when skeletal shape is established, and provide the first demonstration that Fat4 and Dchs1 establish polarized cell behaviour intrinsically within the mesenchyme. Our data also reveal the first indication that cell intercalation processes occur during ventral body wall elongation and closure.

## Abstract (web summary)

How the shape of the sternum is regulated is unclear. Here, the authors identify the Dchs1-Fat4-planar cell polarity pathway as controlling cell orientation and cell intercalation of mesenchymal cells that form skeletal condensations for the mouse sternum, which defines the relative dimensions of the sternum.

## Graphical abstract

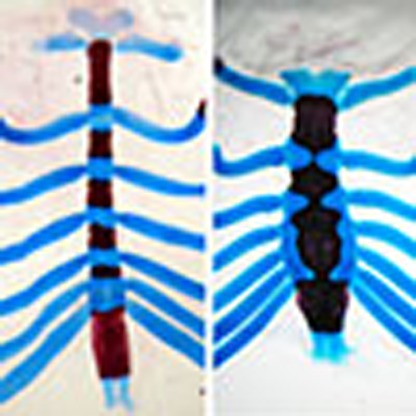

Planar cell polarity (PCP), the co-ordinated behaviour and polarity of cells within the plane of a tissue, is essential for the appropriate morphogenesis, and ultimately function of organs and tissues^1^. In *Drosophila*, PCP regulates cell behaviour within an epithelium and is controlled by two distinct pathways: Fz–Vang PCP, which includes the asymmetrically distributed Fz and Vang proteins, and Ds–Fat PCP, which includes the asymmetrically distributed Fat and Dachsous (Ds) proteins^1^,^2^. These PCP pathways are conserved in vertebrates, where Dchs1 is a homologue of Ds and Fat4 is the homologue of Fat. However, while there are now many examples of cellular polarization in vertebrates controlled by Fz–Vang PCP in both the epithelium and mesenchyme, only two clear examples of cellular polarization directed by Ds–Fat PCP in vertebrates have been identified: Dchs1–Fat4 regulate the orientation of cell divisions within the kidney tubule epithelium and the collective migration of facial branchiomotor neurons within the hindbrain^3^,^4^.

Fat and Ds are large cadherin family proteins that engage in heterophilic binding between neighbouring cells. In *Drosophila*, gradients of Ds and Four-jointed expression result in different levels of Ds–Fat activity on opposing sides of each cell within the gradient. This asymmetry, in turn, establishes polarized cell behaviour, and the gradients ensure that the cells are collectively polarized. Mice mutant for *Fat4* or *Dchs1* are characterized by a wide range of defects in organogenesis, but in general, the underlying mechanisms that cause these defects are unknown^3^,^5^. These abnormalities include changes in the development of the sternum and vertebrae that are endochondral bones forming via a cartilage intermediate.

Development of the endochondral skeleton involves stepwise formation of the pre-chondrogenic mesenchyme, and commitment towards the chondrogenic lineage through the expression of the transcription factor Sox9. The developing chondrocytes become round and secrete matrix, and the cartilage condensation becomes surrounded by a fibroblastic layer, the perichondrium^6^. Skeletal shape is determined during these early phases of morphogenesis, but the molecular networks and processes that determine skeletal shape are in general unknown. In chicks and mice, Fz-PCP signalling has been shown to regulate the orientation of cells within the developing digit condensations, and the integration of proliferating chondrocytes into columnar stacks within the growth plate of long bones^7^,^8^,^9^, but whether Dchs1–Fat4 PCP polarizes cells within the skeletal mesenchyme is unknown.

The sternum arises within the lateral plate mesoderm, and in mice and chicks, the sternal progenitors have been identified in histological sections as a ‘stream of flattened cells' that condense at the tip of the ribs (Fig. 1a)^10^,^11^,^12^. Sternal progenitors are first molecularly detectable around E12.5 within the closing body wall at the tip of the ribs by the expression of chondrogenic/osteogenic markers, *Sox9*, *Runx1 and Runx2* (refs 13, 14). By E15.5 the two sternal bands have met, overt chondrocyte differentiation is apparent, as characterized by alcian blue staining, and the perichondrium has formed (Fig. 1a). Ossification has occured by E16.5 and by P0, the fully differentiated sternum has formed (Fig. 1a). How sternum shape is established is unknown.

Here we show that Dchs1–Fat4 signalling acts via PCP to control polarized cell behaviours within the mesenchyme during the early phases of sternum morphogenesis. In wild-type embryos, sternal progenitors intercalate across the medio-lateral axis simultaneously narrowing the width of the sternum, while increasing its length and depth. These cell intercalation events are typical of convergent-extension processes that are regulated by Fz-PCP in other regions of the embryo. We show that these polarized cell behaviours are established by differential Dchs1–Fat4 expression and that mosaic ablation of Dchs1 disrupts cell polarity. This is the first demonstration that Dchs1–Fat4 regulate PCP within the mesenchyme and is the first evidence that cell intercalation occurs during ventral body wall development.

## Results

### Fat4 and Dchs1 regulate early phases of sternum development

**Figure 1:**
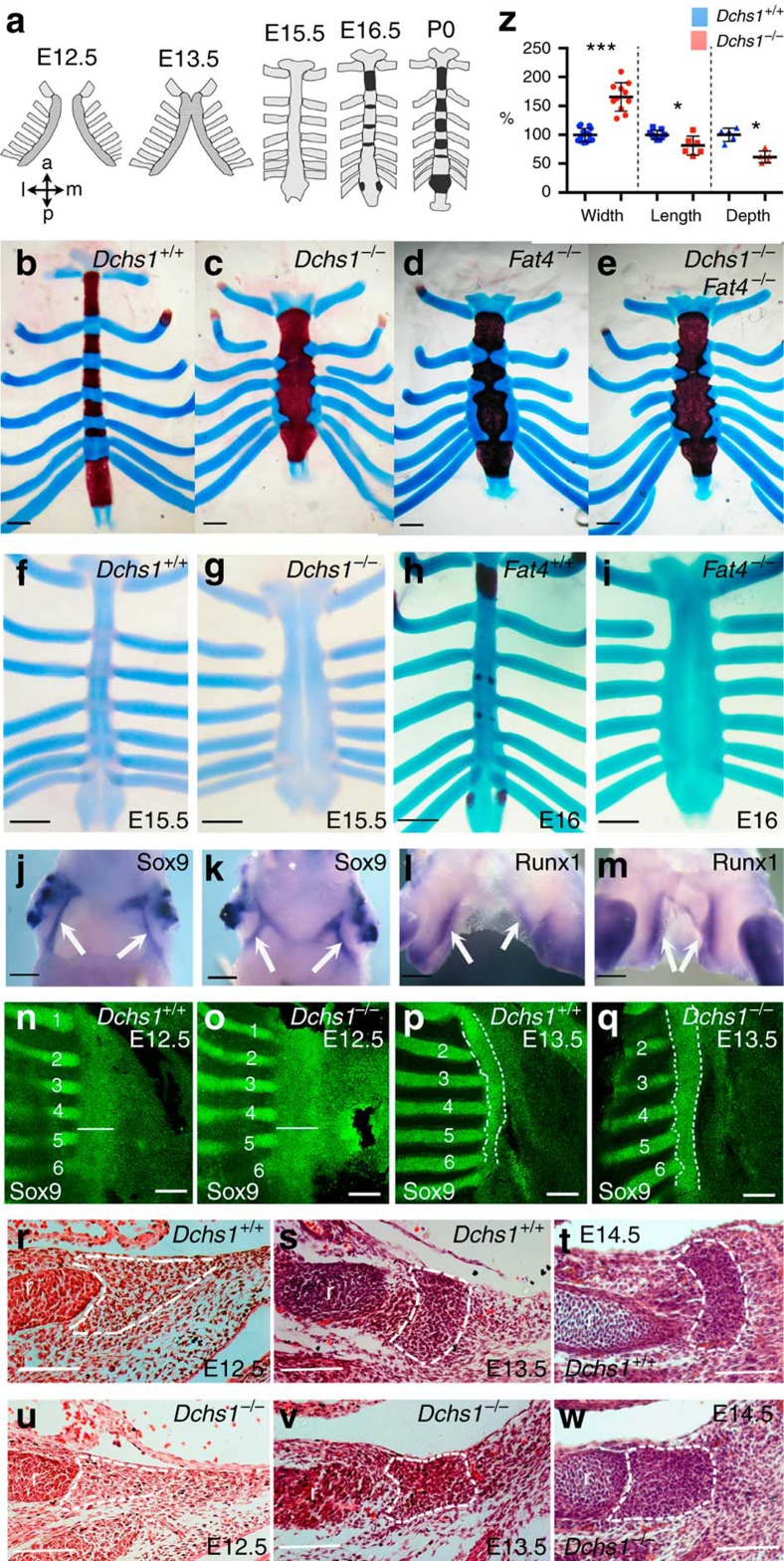
Sternum development in Fat4 and Dchs1 mouse mutants. (**a**) Diagram illustrating mouse sternum development between E12.5 and P0; the darker areas at E12.5 and E13.5 show the pre-cartilaginous condensations and the black regions at E16.5 and P0 indicate ossification of the intercostal regions. (**b**–**i**) Alcian blue and alizarin staining of P0 (**b**–**e**), E15.5 (**f**,**g**), E16.0 (**h**,**i**) sterna and ribs in wild-type (**b**,**f**,**h**), *Dchs1*^*−/−*^ (**c**,**g**), *Fat4*^*−/−*^ (**d**,**i**) and *Dchs1*^*−/−*^*Fat4*^*−/−*^ (**e**) mutants; cartilage is stained blue, bone is red. (**j**–**m**) Ventral views of E12.5 wild type (**j**,**l**), *Dchs1*^*−/−*^ (**k**) and *Fat4*^*−/−*^ (**m**) sterna showing *in situ* hybridizations of Sox9 (**j**,**k**) and Runx1 (**l**,**m**) expression; the sternal plates are arrowed. (**n**–**q**) Flatmounts of E12.5 (**n**,**o**) and E13.5 (**p**,**q**), wild type (**n**,**p**) and *Dchs1*^*−/−*^ (**o**,**q**) sterna that have been immunostained for Sox9 (green), which is expressed in the sternal plate and ribs: the width of the sternal plate is indicated by the white bar in (**n**,**o**) and the sternum is outlined in (**p**,**q**); the ribs are numbered. (**r**–**w**) Haematoxylin and eosin stained sections through the dorso-ventral axis of the developing sternum at E12.5 (**r**,**u**), E13.5 (**s**,**v**) and E14.5 (**t**,**w**) in wild-type (**r**–**t**) and *Dchs1*^*−/−*^ (**u**–**w**) embryos; the sternum is outlined by the white dashes. r, rib. (**z**) Bar chart showing % change in width, length and depth in E14.5 *Dchs1*^*−/−*^ sterna compared with average sterna measurement in wild-type embryos that was standardized to 100%. The lines indicate mean (thicker black bar), 25 and 75%. Student's *t*-test: *P<0.05, ***P<0.001. Scale bars, 1 mM (**b**–**e**,**j**–**m**); 500 μM (**f**–**i**); 200 μM (**n**–**q**); 100 μM (**r**–**w**).

One of the most striking phenotypes of *Dchs1* and *Fat4* null mutant animals is the broadening and shortening of the sternum (Fig. 1b–d). The *Dchs1* and *Fat4* null sternal phenotypes are similar to each other, and are not exacerbated in *Fat4*^*−/−*^ *Dchs1*^*−/−*^ double mutants suggesting that they act as a dedicated signalling pair (Fig. 1b–e)^5^. *Fat4*^*+/−*^ or *Dchs1*^*+/−*^ heterozygotes, and *Fat4*^*+/−*^ *Dchs1*^*+/−*^ double heterozygotes, have normal sternums (Supplementary Fig. 1o,p)^5^.

To determine when the sternum defect arises in *Fat4*^*−/−*^ and *Dchs1*^*−/−*^ mutants, sternum development was characterized from E12.5, when the sternum is specified, until E16.5 when the bone and cartilaginous template has formed. Whole-mount and immunolocalisation studies showed that the chondrogenic and osteoblastic markers *Sox9* and *Runx1* are expressed in sternal precursors, indicating that the sternum is specified appropriately (Fig. 1j–m; Sox9: *Dchs1*^*−*/*−*^, E12.5, *n*=3; Runx1: *Fat4*^*−*/*−*^, E12.5, *n*=2; E13.5, *n*=2). However, whole-mount alcian blue staining of the cartilage matrix, together with histological analyses, revealed obvious changes in the sternum dimensions by E14.5 (Fig. 1t,w; Supplementary Fig. 1e,f). This included an obvious change in the depth of the sternum across the dorso-ventral axis (Fig. 1t,w). Comparison of E14.5 *Dchs1*^*−/−*^ mutants with wild-type littermates identified an average increase of 58% in width (Fig. 1t,w,z; Supplementary Fig. 1s; *P*<0.001; *Dchs1*^*+/+*^/*Dchs1*^*+/−*^, *n*=16 and *Dchs1*^*−/−*^, *n*=11), an average decrease of 39% along the dorso-ventral axis (Fig. 1t,w,z; Supplementary Fig. 1s; *P*<0.0005; *Dchs1*^*+/+*^, *n*=5 and *Dchs1*^*−/−*^, *n*=5) and an average decrease in length of 11% along the anterior-posterior axis (Fig. 1z; *P*<0.05; *Dchs1*^*+/+*^, *n*=11 and *Dchs1*^*−/−*^, *n*=6). Changes in all these dimensions are still apparent at P0 (Fig. 1b–d; Supplementary Fig. 1c,d). Thus, by E14.5 in mice, the template and shape of the post-natal sternum has been established and Dchs1–Fat4 signalling is required during this phase of development.

Analysis of chondrocyte cell density and differentiation at E14.5 revealed no significant changes between wild-type and *Dchs1*^*−/−*^ mutant embryos (Fig. 1t,w; Supplementary Figs 1t and 2c–g; *Dchs1*^*+/+*^, *n*=3 and *Dchs1*^*−/−*^, *n*=4). The changes in sternum shape must, therefore, be due to an altered arrangement of chondrocytes and cannot be due to differences in chondrocyte differentiation, such as reduced hypertrophy. However, there are changes in the timing and pattern of ossification of the sternum at later stages of development in mutants, which can be attributed to the altered dimensions of the sternum. In the wild-type sternum, ossification is inhibited by signals from the ribs that results in the formation of the separate intercostal regions^15^. Reflecting the alteration in sternum morphology, the timing of ossification was slightly delayed at E16.5 presumably due to the proximity of the ribs to each other in *Fat4*^*−/−*^ and *Dchs1*^*−/−*^ mutants (Fig. 1h,i; Supplementary Fig. 1a,b). By P0, the sternum was almost fully ossified in *Fat4*^*−/−*^ and *Dchs1*^*−/−*^ mutants with only discrete regions adjacent to the ribs remaining cartilaginous, which is again consistent with an inhibitory ossification signal from the ribs (Fig. 1c,d).

Immunohistological analysis of sternal flatmounts and tissue sections together with histological analyses showed there is a subtle increase in width at E12.5, although no obvious histological changes were identifiable and the sternal progenitors were found loosely organized at the tip of the rib in both wild-type and *Dchs1*^*−/−*^ or *Fat4*^*−/−*^ embryos (Fig. 1n,o,r,u; Supplementary Fig. 1g,h,k,l). The phenotype is, however, clearly apparent by E13.5: in *Dchs1*^*−/−*^ and *Fat4*^*−/−*^ embryos the sternal condensation is wider along the medio-lateral axis and shorter along the dorso-ventral axis when compared with wild-type or heterozygous littermates (Fig. 1p,q,s,v; Supplementary Fig. 1i,j,m,n,s). Furthermore, unlike in wild-type embryos, the sternal bar does not significantly narrow between E13.5 and E14.5 (Fig. 1s,t,v,w; Supplementary Fig. 1s; wild type, *P*<0.05; *Dchs1*^*−/−*^ not significant). These defects are before the formation of the perichondrium and overt chondrocyte differentiation as characterized by alcian blue staining and therefore occur within the early-condensing mesenchyme. In contrast, early development of the limb skeletal elements, which are also derived from the lateral plate mesoderm, is unaffected (Supplementary Fig. 1q,r; *Fat4*^*−/−*^, *n*=4).

### Cell polarity is altered in Fat4 and Dchs1 mutants

The phenotype emerges within the condensing mesenchyme preferentially affecting the medio-lateral and dorso-ventral axes. In wild-type embryos, the sternum narrows between E13.5 and E14.5, while increasing in length along the dorso-ventral axis (Fig. 1s,t; Supplementary Fig. 1s), suggestive of polarized cell behaviours, such as those occuring during convergent extension^16^,^17^. Convergent extension describes the intercalation of cells towards each other, which narrows the width of the tissue while simultaneously increasing the length and/or thickness. Hallmarks of convergent extension within the gastrulating mesenchyme in *Xenopus*, where this process has been well characterized, include the flattening and elongation of cells across the medio-lateral axis and the formation of extended filopodia at the medial and lateral edges of the cells^17^,^18^,^19^. Orientated cell divisions (OCD) can also contribute to directed tissue growth by directing the cell divisions across one axis of the tissue. If convergent extension and OCD contribute to sternum narrowing and thickening in wild-type embryos, we would expect cells to be orientated along the medio-lateral axis to allow cell intercalation and cell divisions to be directed along the dorso-ventral axis, which would increase the depth of the sternum.

**Figure 2:**
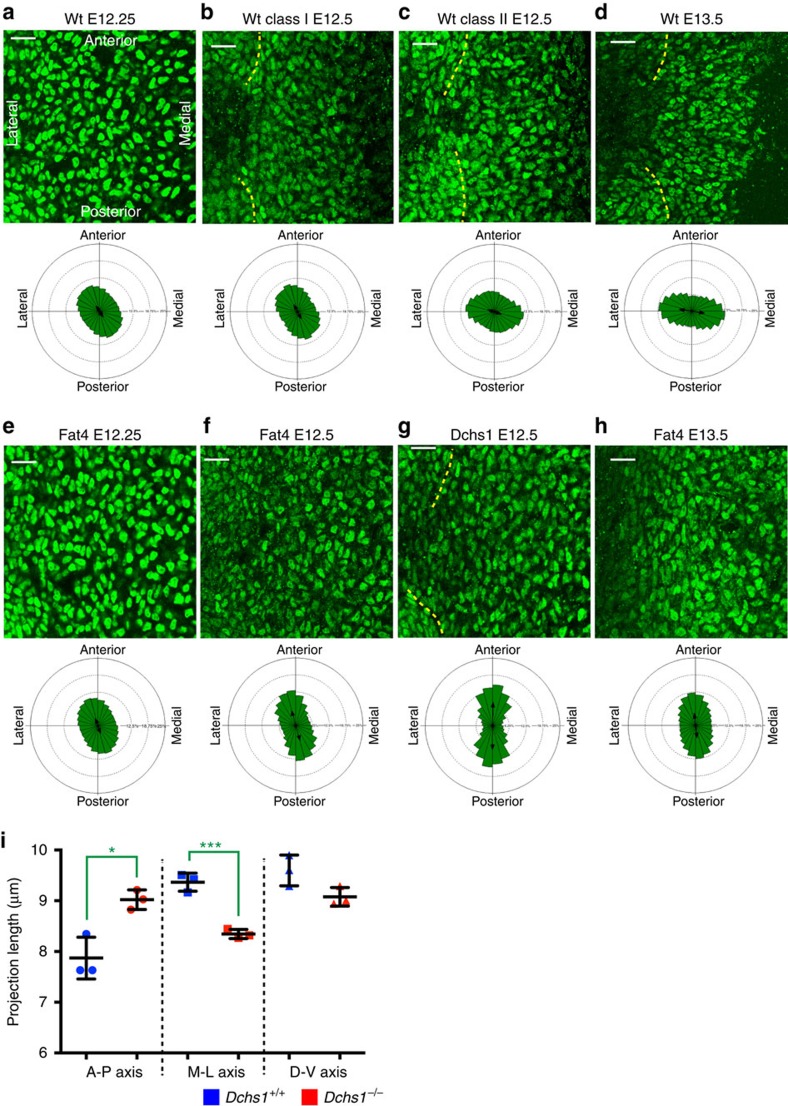
Analysis of sternal cell nuclear orientation in wild type and in mutants. (**a**–**h**) Flatmounts of E12.25 (**a**,**e**), E12.5 (**b**,**c**,**f**,**g**) and E13.5 (**d**,**h**), from wild type (**a**–**d**), *Dchs1*^*−*/*−*^ (**g**) and *Fat4*^*−*/*−*^ (**e**,**f**,**h**) sterna immunostained for Sox9 (green) to mark sternal nuclei. Orientation of the nuclei is shown in the rose plots below. The lateral edges of the sterna are indicated by dashed lines. The anterior-posterior and medio-lateral axes are defined in **a**. The total numbers of cells analysed are as follows: wt; E12.25, 40,569; E12.5, class I 15,837; E12.5 class II, 23,149; E13.5, 46,042; *Fat4*^*−/−*^, E12.25, 40,468; E12.5, 30,443; E13.5, 59,152; *Dchs1*^*−/−*^, E12.5, 2,484; E13.5, 2,575. (**i**) Quantitation of projection length of nuclei along anterior-posterior (A-P), medio-lateral (M-L) and dorso-ventral (D-V) axes (see Supplementary Fig. 3a–f for an example of the analysis; *n*=3, >1,500 cells analysed per embryo). The lines indicate mean (thicker black bar), 25 and 75%. Student's *t*-test: *P<0.05, ***P<0.001. Scale bar, 25 μM.

Cell orientation and polarity were initially examined by determining the direction of the long axis of the nuclei in flatmounts of E12.5 and E13.5 sterna, as nuclear orientation can indicate cell polarity, and can be more easily scored. In wild-type E13.5 embryos, nuclei were preferentially orientated along the medio-lateral axis (Fig. 2d; *n*=18; mean vector 171.4°; Rao's spacing test, *P*<0.05). In E12.5 embryos, nuclear orientation was more variable, but we found embryos could be divided into two classes. In some E12.5 embryos (class I), the nuclear long axis was preferentially orientated along an axis closer to anterior-posterior than medio-lateral, whereas in others (class II) the nuclear orientation was close to random with a slight bias towards the medio-lateral axis (Fig. 2b,c; class I, *n*=7, mean vector 119.2°; class II, *n*=7, mean vector 161.5°). This suggested that cells might be undergoing a dynamic transition during E12.5 to E13.5 from a more anterior-posterior orientation towards a preferentially medio-lateral orientation. Consistent with this interpretation, when we analysed E12.25 embryos, nuclear orientation resembled that of class I E12.5 embryos (Fig. 2a; *n*=8, mean vector 123.9°). Analysis of mutant embryos revealed that this shift towards medio-lateral orientation does not occur (Fig. 2e–h). At E12.25, nuclear orientation in *Fat4* mutant embryos was similar to that in wild type (Fig. 2e, *n*=4, mean vector 109.5°). At E12.5 and E13.5, a preferentially anterior-posterior orientation of nuclei became even more evident in both *Dchs1* and *Fat4* mutant embryos that was significantly different from nuclear orientation in wild-type embryos (Fig. 2f–h, E12.5: *Fat4*^*−/−*^ *n*=10, mean vector 106.3°, *P*<0.01; *Dchs1*^*−/−*^ *n*=4, mean vector 89.1°, *P*<0.01; E13.5: *Fat4*^*−/−*^ *n*=15, mean vector 95.7°, *P*<0.01; *Dchs1*^*−/−*^ *n*=5, mean vector 84.3° *P*<0.01; Mardia–Watson–Wheeler test at E12.5 and E13.5, *P*<10^−12^). Analysis of the cell orientation in tissue sections through the dorso-ventral axis also showed that sternal cells are polarized in wild-type embryos but not in *Dchs1*^*−/−*^ (Mardia–Watson–Wheeler test, *P*=10^−12^) or *Fat4*^*−/−*^ (*P*=6 × 10^−6^) mutants (Supplementary Fig. 3g; wild type, *n*=4; *Dchs1*^*−/−*^*, n*=2; *Fat4*^*−/−*^*, n*=2). The difference between wild type and mutants is not simply due to developmental delay, because even at E14, mutant nuclei are still aligned along the anterior-posterior axis (Supplementary Fig. 2a).

To analyse nuclear alignment in three-dimensions, confocal *z*-stacks were used to generate reconstructions of the nuclei in the wild-type and *Dchs1*^*−/−*^ embryos. The orientation and length of the nuclei along the three axes, together with the shape (sphericity) and total nuclear volume, were then determined. This revealed that the mean sphericity of nuclei was comparable in *Dchs1*^*+/+*^ and *Dchs1*^*−/−*^ sterna (0.8775±0.0032 for *Dchs1*^*+/+*^ *n*=3 and 0.8798±0.0036 for *Dchs1*^*−/−*^ *n*=3, *P*=0.6395) and the average volumes of nuclei analysed also did not differ (277.9±13.28 μm^3^ in *Dchs1*^*+/+*^ and 274.7±8.67 in *Dchs1*^*−/−*^, *P*=0.847). However, as shown in the previous analysis, the orientation of the nuclei did differ. This was revealed by projecting nuclei onto the anterior-posterior, medio-lateral and dorso-ventral axes (Supplementary Fig. 3a–f). In wild-type embryos, nuclear lengths are longer along the medio-lateral axis (mean 9.37±0.10 μm) than the anterior-posterior axis (mean 7.87±0.24 μm; Fig. 2i; Supplementary Fig. 3a,b). Conversely, in *Dchs1*^*−/−*^ embryos a preferential alignment along the anterior-posterior axis versus the medio-lateral axis was observed (mean 9.02±0.11 versus a mean 8.34±0.52 μm along the medio-lateral axis; Fig. 2i; Supplementary Fig. 3d,e). There were no significant differences in the length of the nuclei along the dorso-ventral axis (Fig. 2i; Supplementary Fig. 3c,f).

**Figure 3:**
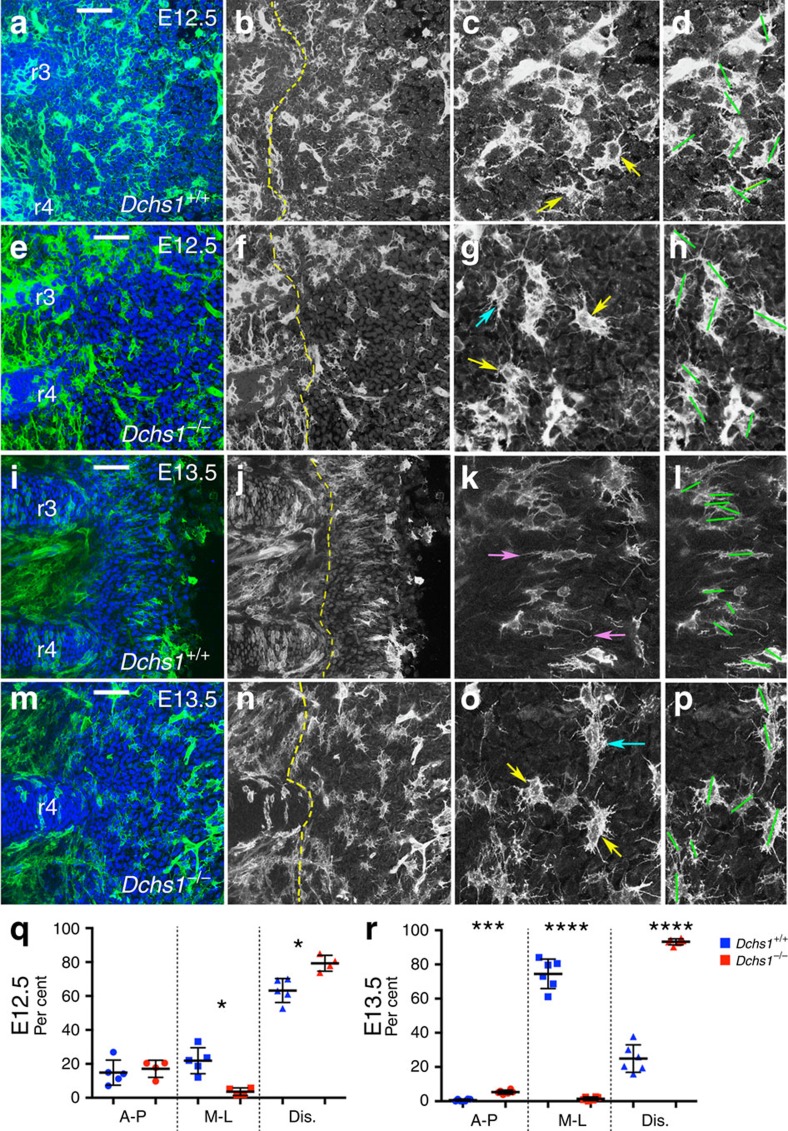
Dchs1^*−*/*−*^ mutant sternum cells are unable to polarize. (**a**–**p**) Flatmounts of *Dchs1*/mT/mG wild-type (**a**–**d**,**i**–**l**) and *Dchs1*^*−*/*−*^/mT/mG mutant (**e**–**h**,**m**–**p**), E12.5 (**a**–**h**) and E13.5 (**i**–**p**) sterna that have been treated with tamoxifen to induce mosaic expression of GFP. The boundary of the ribs are indicated by the yellow dashed lines in **b**,**f**,**j** and **n**. The rib number is indicated (see Fig. 1n–q for numbering of the ribs). (**a**,**b**,**e**,**f**,**i**,**j**,**m**,**n**) show low-magnification views where (**b**,**f**,**j**,**n**) are the black and white images of **a**,**e**,**i** and **m**, respectively. (**c**,**d**,**g**,**h**,**k**,**l**,**o**,**p**) show high-power images; the long axis of the cells as determined by the orientation of the nuclei is indicated by the green lines in **d**,**h**,**l** and **p**. For analysis of the filopodia orientation, a series of *z*-projections of 50 slices each (0.17 μm steps) were prepared in Fiji for each scan and cells scored based on the GFP signal demarcating the cell outlines and filopodia. Two classes of cells were noted: cells with long bipolar extensions and cells with multiple filopodia lacking defined orientation. The bipolar cells were further classified depending on the alignment of the filopodia along the medio-lateral or dorso-ventral axes of the sternum. Examples of these groups are shown in **c**,**g**,**k** and **o**. Yellow arrows indicate cells with multiple random projecting filopodia, pink arrows show cells with filopodia predominantly projected across the medio-lateral axis and blue arrows show cells where the filopodia are extended across the anterior-posterior axis. (**q**,**r**) Quantification of the % of cells showing randomly projecting filopodia (Dis) or filopodia that are predominantly projected along the anterior-posterior (A-P) or medio-lateral (M-L) axis at E12.5 (**q**) and E13.5 (**r**). The lines indicate mean (thicker black bar), 25 and 75%. The number of cells analysed are as follows: E12.5, *Dchs1*^*+/+*^ (945), *Dchs1*^*+/−*^ (862), *Dchs1*^*−/−*^ (910); E13.5, *Dchs1*^*+/+*^ (1,221), *Dchs1*^*−/−*^ (1,923); significance analysed by the Student's *t*-test: *P<0.05; ***P<0.0005; ****P<0.0001. Scale bar, 100 μM.

To analyse the polarity and direction of the filopodia of sternal cells, another critical indicator of convergent extension, Dchs1/mT/mG mice were generated. In these mice, mosaic green fluorescent protein (GFP) expression can be induced by tamoxifen induction of Cre-recombinase allowing the visualization of isolated cells. The direction of the filopodia was scored and cells were assigned into the following three categories: cells that had filopodia that were predominantly directed along the medio-lateral (M-L) axis, the anterior-posterior (A-P) axis or that had multiple randomly orientated protrusions. At E12.5 in both *Dchs1*^*+/+*^ and *Dchs1*^*−/−*^ mutants, the majority of the cells were oriented along the anterior-posterior axis and had multiple filopodia that projected randomly (Fig. 3a–h; *Dchs1*^*+/+*^, *n*=3; *Dchs1*^*−/−*^, *n*=3). The latter is characteristic of unpolarized mesenchymal cells. Nonetheless, differences in cell behaviour start to emerge at this stage as in *Dchs1*^*+/+*^ embryos significantly fewer cells had multiple randomly projected filopodia compared with *Dchs1*^*−/−*^ embryos (Fig. 3q; *P*<0.05, *n*=3). Conversely, significantly more cells in *Dchs1*^*+/+*^ embryos had filopodia that were directed along the medio-lateral axis when compared with *Dchs1*^*−/−*^ mutants (Fig. 3q; *P*<0.05, *n*=3). By E13.5, there were striking differences: in *Dchs1*^*+/+*^ sterna the majority of cells had filopodia orientated along the medio-lateral axis (Fig. 3i–k,r; *n*=3), whereas in *Dchs1*^*−/−*^ embryos, the majority of cells still had multiple randomly projected filopodia (Fig. 3m–o,r; *P*<0.0001). More cells in *Dchs1*^*−/−*^ sterna also had projections that were predominantly directed along the anterior-posterior axis when compared with wild-type embryos (Fig. 3r, *P*<0.0005).

In wild-type embryos at E13.5, sternal cells are also very closely packed and aligned across the medio-lateral axis (Fig. 3k,l): this ‘alignment' of cells across the medio-lateral axis was also observed in the sections through the dorso-ventral axis at E13.5 and E14.5 (Supplementary Figs 2c,e,f and 3h). Analysis of the actin cytoskeleton at E13.5 also revealed a preferential alignment of F-actin across the medio-lateral axis of the wild-type cells (Supplementary Fig. 4a,c; *Fat4*^*+/+*^, *n*=3). In contrast, cells and the F-actin cytoskeleton are less organized in *Dchs1* and *Fat4* mutants (Fig. 3o,p; Supplementary Figs 2d,g, 3i and 4b,d). In conclusion, in wild-type sternal cells, filopodia and the actin cytoskeleton become polarized, which in turn would be expected to alter cell shape, orientation and the relative nuclear dimensions.

### Evidence of cell intercalation in the developing sternum

**Figure 4:**
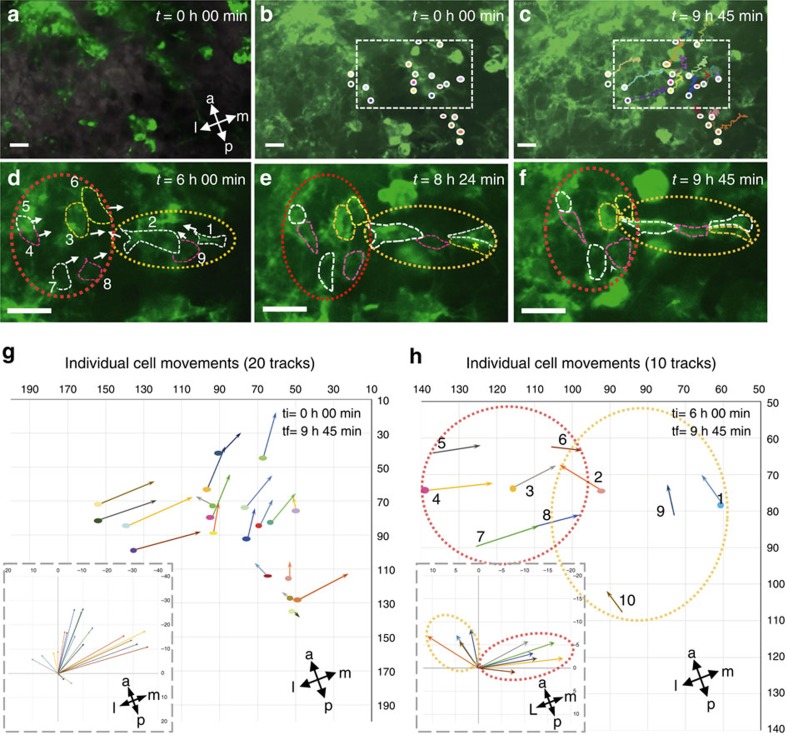
Evidence for cell intercalation movements in a developing E12.5 sternum. (**a**–**c**) Low-power image of time zero (**a**,**b**) and time 9 h 45 min (**c**) of a time lapse movie of a developing E12.5 mTmG sternum cultured as a flatmount. A few cells express GFP following tamoxifen induction at E10.5. The movement of 20 cells along the medio-lateral and anterior-posterior axes between these time points is shown in **b**,**c** and is illustrated graphically in **g**. In **g** and **h**, each cell is indicated by a different colour arrow. The main image shows the relative movement of cells to each other. The graph on the bottom left hand side shows the relative movement of cells around the medio-lateral and anterior-posterior axes. Most cells move either medially or anteriorly due to tissue growth and ‘drifting' during the imaging. The short trajectory lines in **g** indicate that the cell had moved in or out of the plane of focus during imaging (along the D-V axis). (**d**–**f**) are high-power views of the boxed region in **b**,**c** at different time points showing two populations of cells that move closer to each other. The red dashed oval shows a group of cells that moves from lateral to medial; the yellow dashed oval shows a second group of cells that moves in the opposite direction from medial to lateral. (**h**) shows a graph illustrating their movements from 6h to 9h 45 min. There are 10 cells within these groups: each cell is outlined by dashed lines and is numbered in **d**,**h**. Scale bars, 17 μm.

The above data are all consistent with cells undergoing convergent extension^17^ and therefore, we also attempted to examine the potential for convergent extension within *ex vivo* sternal cultures. We were able to identify examples of individual cells that move towards each other at E12.5 in a manner consistent with the inference of convergent extension (Fig. 4). These cell movements would be predicted to decrease the width of the developing sternum along its medio-lateral axis.

### Orientated cell divisions do not contribute to sternum shape

A combination of convergent extension and OCD are needed for the elongation of other embryonic structures, such as the kidney tubules and neural tube^17^,^19^. If OCD contribute to the morphogenesis of the sternum, we would predict that cell divisions would be directed along the dorso-ventral axis of the sternum, and in mutant sterna, this directed cell division would be lost or re-orientated along the medio-lateral axis. To determine the orientation of cell divisions, 4,6-diamidino-2-phenylindole staining was used to identify cells at terminal anaphase/early telophase (Supplementary Fig. 5e). Five cells were identified (0.045%; 11,000 cells analysed), but there was no obvious bias in the orientation of cell division in wild-type embryos (Supplementary Table 1). Analysis of *Dchs1*^*−/−*^ and *Fat4*^*−/−*^ mutants also indicated that there is no predominant bias in the orientation of cell divisions (Supplementary Table 1). No OCD were identified at E13.5 either (Supplementary Table 1). This analysis indicates that OCD are very unlikely to contribute to the morphogenesis of the wild-type sternum and that an alteration in OCD cannot explain the widened sternum *in Dchs1*^*−/−*^ and *Fat4*^*−/−*^ mutants.

### Fat4–Dchs1 act independently of Hippo effectors Yap/Taz

In *Drosophila*, Fat–Ds regulate PCP and also inhibit the transcriptional co-activator Yorkie, by promoting Hippo signalling, which regulates cell proliferation, survival, migration and differentiation^20^,^21^,^22^. There is some evidence that the Fat–Hippo pathway may be conserved in vertebrates although this is controversial^23^,^24^,^25^,^26^,^27^. We have already shown that chondrocyte differentiation is unchanged, but to further examine potential interactions between the Dchs1–Fat4 and Hippo pathways, alterations in cell proliferation and cell death were analysed at E12.5 and E13.5 by immunostaining for phospho-histone H3 and activated caspase 3, respectively. No significant changes in proliferation were identified (E12.5, *Dchs1*^*−/−*^, *n*=4; *Fat4*^*−/−*^, *n*=3; E13.5, *Dchs1*^*−/−*^, *n*=3; Supplementary Fig. 5a–c). The occasional apoptotic cell was identified around the edge of the cartilage condensation in both wild-type and mutant sterna at E12.5 and E13.5, but no obvious differences were observed (Supplementary Fig. 5b,c). To further exclude Dchs1–Fat4 regulation of Hippo signalling during sternum morphogenesis, we generated double Fat4^*−*/*−*^/Taz^*−*/*−*^ and Fat4^*−*/*−*^/Yap^+/*−*^ mouse mutants. Taz and Yap are the transcriptional effectors of the vertebrate Hippo pathway. If loss of Fat4/Dchs1 mediate their effects through the activation of Yap and Taz, sternum development could be partially rescued in Fat4^*−*/*−*^/Taz^*−*/*−*^ and/or Fat4^*−*/*−*^/Yap^+/*−*^ mutants. However, we found that the sterna remained shorter and wider following the reduction or deletion of Yap and Taz, respectively (Supplementary Fig. 5f–h; Fat4^*−*/*−*^/Yap^+/*−*^, *n*=4; Fat4^*−*/*−*^/Taz^*−*/*−*^ *n*=5). Collectively, these data indicate that alterations in sternum morphogenesis are unlikely to be due to effects of Dchs1 and Fat4 on Yap and Taz^24^,^28^.

### Gradients of Fat4 and Dchs1 determine cell polarity

**Figure 5:**
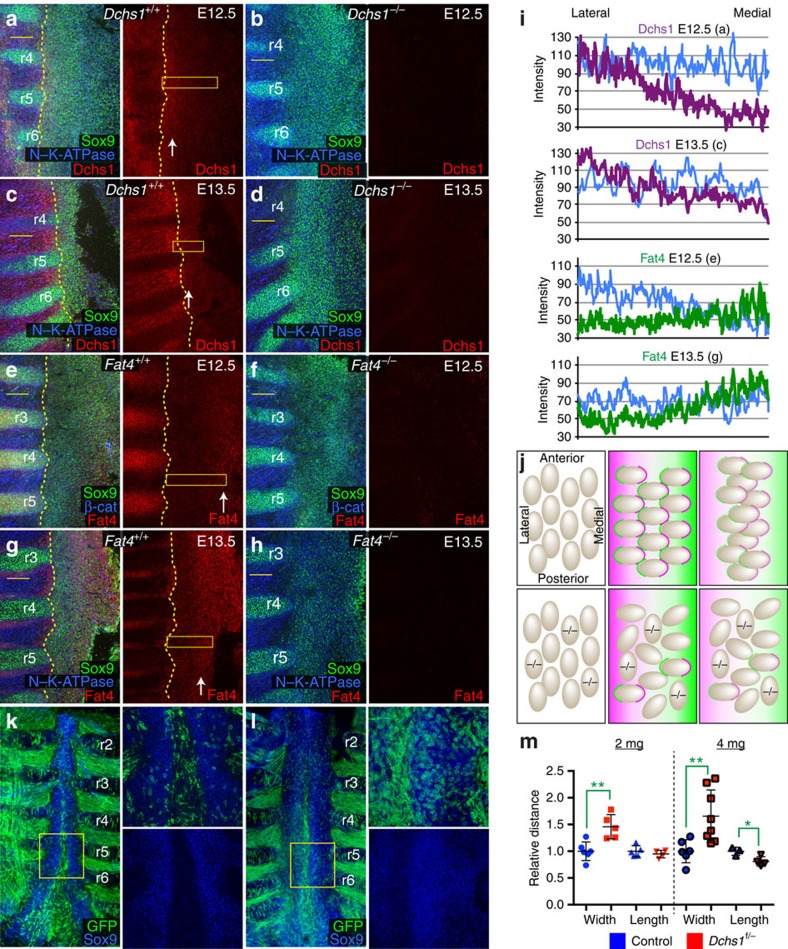
Differential expression of Fat4 and Dchs1 across the medio-lateral axis of the sternum controls sternum morphogenesis. Flatmounts of E12.5 (**a**,**b**,**e**,**f**) and E13.5 (**c**,**d**,**g**,**h**) sterna immunostained for expression of Sox9 (**a**–**h**), N–K-ATPase (**a**–**d**,**g**,**h**), β-catenin (**e**,**f**) and Dchs1 (**a**–**d**) or Fat4 (**e**–**h**). (**a**,**c**,**e**,**g**) wild-type sterna; (**b**,**d**) and (**f**,**h**) show *Dchs1*^*−/−*^ and *Fat4*^*−/−*^ mutant sterna, respectively, to determine the levels of background antibody staining. r, rib. White arrows indicate regions of expression. Boundary of sternum marked by yellow dashed lines. (**i**) Graphs showing levels of Dchs1 (magenta) or Fat4 (blue) across the medio-lateral axis in the boxed region of the sternum (**a**,**c**,**e**,**g**): the blue line indicates the levels of N–K-ATPase or β-catenin, which were used as ‘standards' for potential changes in cell density. (**k**,**l**) E14.5 sternums with mosaic deletion of Dchs1 following 4 mg tamoxifen treatment at E10.5; the extent of mosaicism indicated by expression of GFP (green) and sternal nuclei stained for Sox9 (blue). (**k**) Wild type and (**l**) Dchs1^f/*−*^. (**j**) Schematic illustration of model for cell orientation and polarization by Dchs1–Fat4. Top left: cells oriented along anterior-posterior axis at E12.25. Top middle: cells become polarized along the medio-lateral axis by differential expression of Fat4 (green), which is highest medially, and Dchs1 (magenta), which is highest laterally. On the basis of the studies in *Drosophila*, this differential expression of Fat4 and Dchs1 is hypothesized to lead to intracellular polarized localization of Dchs1 and Fat4, which determines cell polarity. Top right: cells intercalate along their axis of polarization, resulting in a narrowing and thickening of the sternum together with an increase in length along the anterior-posterior axis. Bottom middle panel: in a Dchs1 mosaic, mutant cells (−/−) are unable to polarize and reorient, and also disrupt the polarization and orientation of neighbouring cells. Bottom right: consequently cells fail to undergo directed intercalation, and the sternum remains wide and flat. (**m**) Histogram summarizing relative increase in width and decrease in length in sterna with mosaic deletion of Dchs1, generated through treatment with 2 or 4 mg tamoxifen. The lines indicate mean (thicker black bar), 25 and 75%. Significance analysed by Student's *t*-test, *P<0.05, **P<0.01. Scale bars, 100 μM.

We have previously shown that Fat4 and Dchs1 are expressed in opposing gradients across the hindbrain and that cells become polarized along these gradients^4^. This is reminiscent of the situation in *Drosophila*, in which gradients of Ds and a modulator of Ds–Fat binding, Four-jointed, direct Ds–Fat PCP^2^. To examine whether differential expression of Fat4 and Dchs1 could contribute to PCP in the sternum, the expression of Fat4 and Dchs1 was determined by immunolocalisation at E12.5 and E13.5, and their expression levels were quantified against either N–K-ATPase or β-catenin (Fig. 5a,c,e,g,i). Background antibody staining was determined by immunostaining *Dchs1*^*−/−*^ or *Fat4*^*−/−*^ sterna as appropriate (Fig. 5b,d,f,h). This analysis revealed that Dchs1 and Fat4 are differentially expressed along the medio-lateral axis of the E12.5 and E13.5 developing sternum, with Dchs1 expression highest laterally in the sternal cells near the ribs, whereas Fat4 expression is highest in sternal cells closest to the ventral midline (Fig. 5a,c,e,g,i). Quantification of the expression levels revealed evidence of opposing gradients of Fat4 and Dchs1 expression (Fig. 5i). These gradients would be expected to establish intracellular polarity within each sternal cell. Cells would have higher levels of Dchs1 on their medial sides where there are higher levels of Fat4 in the adjacent cells and conversely, higher levels of Fat4 on their lateral sides where the Dchs1 expression is highest (Fig. 5j). To confirm that Fat4 functions within the mesenchyme, Fat4^f/f^Dermo1^cre^ were generated to specifically inactivate Fat4 expression within the mesoderm and not the overlying epithelium. Micro-computerized tomography scanning of Fat4^f/f^Dermo1^cre^ P0 mice revealed they have a broadened sternum resembling *Fat4* and *Dchs1* null mice (Supplementary Fig. 6a,b; *n*=3).

We considered two potential models for how Dchs1–Fat4 polarize cells and the filopodia. In one model, Fat4 and Dchs1 may regulate cell adhesion, and cells may extend filopodia preferentially to regions of highest Fat4 and Dchs1 expression. In the second model, Dchs1–Fat4 gradients may establish an intracellular polarity (with different levels of Dchs1 and/or Fat4 on the medial versus lateral axis of the cell), which in turn determines cell polarity. The latter model is based on studies in *Drosophila*, where the establishment of Ds–Fat PCP involves the polarization of Ds and Fat proteins within each cell. Importantly, a cell with polarized Ds and Fat can serve as a template for a similar polarization of Ds and Fat in its neighbours^29^,^30^,^31^. This enables Ds–Fat PCP to spread throughout a tissue without requiring each individual cell to ‘read' gradients of differential Ds expression. A characteristic feature of this type of polarization is that it is easily disrupted by small patches of mutant tissue, because this prevents the propagation of polarization. Indeed, in the hindbrain, we found that having even a small percentage of *Dchs1* mutant cells was sufficient to severely disrupt PCP^4^. In contrast if filopodia extend to regions of highest Fat4 and Dchs1 expression, or Fat4/Dchs1 regulate cell adhesion, this would not be disrupted by the presence of a few mutant cells. To determine whether a gradient of Dchs1 establishes cell polarity during sternum morphogenesis, we generated mosaics in which Dchs1 was deleted in a *Dchs1*^*+/−*^ background. Specifically, we generated Dchs1^f/*−*^/mT/mG embryos in which tamoxifen treatment would produce a mosaic of Dchs1 null GFP-expressing cells in a heterozygous background. These null cells would be predicted to disrupt cellular behaviours both cell and non-cell autonomously (Fig. 5j). The width and length of the sternum were analysed following Sox9 immunostaining at E14.5.

We found that mosaic disruption of Dchs1 following 2 or 4 mg of tamoxifen treatment resulted in an increase in the width of the sternum (Fig. 5k–m; Supplementary Fig. 6c,d). Treatment with 2 mg tamoxifen resulted in an average increase of 56% in width (*n*=6, *P*<0.01), while there was an average increase of 70% following treatment with 4 mg of tamoxifen (Fig. 4m; *n*=9, *P*<0.005). The length of the sternum was also significantly reduced at the higher dose of tamoxifen (Fig. 4m; *P*<0.05). Significantly, a mosaic with as few as 3% GFP cells was 30% wider. These data clearly demonstrate that the presence of a few *Dchs1* null cells causes the loss of polarized cell behaviour autonomously and non-cell autonomously.

## Discussion

Here we have identified a new role for Dchs1–Fat4 PCP in vertebrates by establishing that Dchs1–Fat4 control cell orientation within the sternal mesenchyme to determine its final shape. In the developing sternum, cells polarize along the axis of differential Fat4 and Dchs1 expression, and mosaic disruption of Dchs1 perturbs sternal development, implying that differential expression of Dchs1 and Fat4 polarizes cells. In *Drosophila*, the graded expression of Ds and Fj results in a polarized sub-cellular localization of Ds and Fat, and this molecular polarization is responsible for directing cellular polarization; molecular polarization of Dchs1 protein has also been observed in the developing kidney^26^. We suggest that the polarization of sternal progenitors established by Dchs1 and Fat4 biases cell intercalations as sternal progenitors condense, to achieve the correct shape of the sternum.

Our results establish that Dchs1–Fat4 PCP can control cell orientation and behaviours within the mesenchyme, and that both vertebrate PCP pathways influence the shape and morphogenesis of skeletal structures. In *Drosophila*, Ds–Fat PCP has only been identified within epithelial cells. Dchs1–Fat4 are expressed in both epithelial and mesenchymal cell populations^5^,^32^, and our discovery establishes Dchs1–Fat4 as an important regulator of PCP within both epithelial and mesenchymal cell populations. Thus, like the Fz–Vang PCP pathway, in vertebrates the Fat-PCP pathway has also been co-opted to determine polarized cell behaviours within the mesenchyme in addition to the epithelium. In addition, as previously shown in the hindbrain, we have also shown that differential or gradients of Dchs1–Fat4 within the sternal mesenchyme determine cell polarity. Thus, we provide further support that mechanisms of Ds–Fat PCP are conserved between *Drosophila* and vertebrates. *Dchs1* and *Fat4* are widely expressed in mesenchymal tissues and such effects on cell behaviour are likely responsible for the diverse symptoms in human patients with mutations in DCHS1 or FAT4 (refs 24, 33).

The Fz–Vang PCP pathway controls elongation of the long bones in chicks and mice, and pharyngeal arch cartilages in fish^7^,^8^,^9^,^34^ and our studies here identify Ds–Fat PCP as a critical determinant of sternal shape in mice. Differential or combinational Fz-PCP and Fat-PCP could, therefore, be utilized to generate the different shapes of skeletal structures and may also be modulated to bring about evolutionary change. For example, our data raise the possibility that modulation of Dchs1–Fat4 signalling results in evolutionary adaptations of sternum. These evolutionary differences are particularly notable in avians, where the sternal complex has been modified to accommodate variations in locomotion and predatory behaviours. For example, the sternum can be ‘square', as in ostriches and albatrosses, resembling the murine *Dchs1*^*−/−*^ and *Fat4*^*−/−*^ mutant sterna, or narrower and elongated, as in parakeet and penguins, more closely resembling sternal shape in wild-type mice. Thus, we speculate that modulation of the Dchs1–Fat4 PCP pathway could play a key role in morphological changes during evolution.

## Methods

### Mice

All mouse procedures were approved by the Institutional Animal Care and Use Committee (IACUC) at Rutgers University or Institutional Animal Care or Use Committee of King's College London, UK, in agreement with established guidelines for animal care. The day of the mouse plug was assigned E0.5. The mouse lines used were: Fat4 (ref. 3), Dchs1 (ref. 5), Gt(ROSA)26Sor^tm4(ACTB-tdTomato-EGFP)Luo^ (mT/mG)^35^ and Gt(ROSA)26Sor^tm1(cre/ERT2)Tyj^/J (ref. 36). Mouse embryos were analysed at the ages indicated, without regard to sex. Tamoxifen inducible mosaic embryos in heterozygous background were generated by mating Dchs1^f/f^ R26^mT/mG^ line with Cre-ER^TM^Dchs1^+/*−*^ line. Tamoxifen (2 or 4 mg; Sigma-Aldrich T5648) injections were performed at E10.5.

### Genotyping

For genotyping, DNA was extracted from tail snips or yolk sacs using reagent DirectPCR (Viagen) and Takara Ex-Taq PCR enzyme. Alternatively, DNA was isolated using Direct PCR-Ear Lysis reagent (PeqLab). The genotype was determined by PCR using allele-specific primers. For Dchs1: dchs1-6,232 5′-CCCCCAGACATTCTCAGCCCTTCTTCTA-3′ and dchs1-9,639 5′-GCTGGGCTTACAGTGCTGAGCAATGAT-3′; product sizes: wild type, 3.4 kb; mutant deletion, 0.7 kb; melting temperature, 57 °C. For Fat4 mutant allele: ft4-59,901 5′-GCGGTGTAAGCTCAATCGATCGACCTT-3′ and Neo-1,399 5′-CGCAGCGCATCGCCTTCTAT-3′; product size: mutant deletion, 1.2 kb; melting temperature, 55 °C. For Fat4 wild-type allele: ft4-F 5′-GGTGCCAACGCTCTGGTCACGTATGC-3′ and ft4-R 5′-CAGGGGTTGTGTCTTCTGGGATGTC-3′; product size: wild type, 0.25 kb; melting temperature, 62 °C. For wild-type Rosa26 allele and mT/mG (tomato/GFP) insertion: T-common 5′ CTCTGCTGCCTCCTGGCTTCT-3′, Tomato-mT/mG 5′-TCAATGGGCGGGGGTCGTT-3′ and R26-WT 5′-CGAGGCGGATCACAAGCAATA-3′; product sizes: wild type, 0.33 kb; mT/mG (tomato/GFP), 0.25 kb; melting temperature, 61 °C. For FVB.Cg-Tg(CAG-cre/Esr1*)5Amc: Cre/Esr1 5′-GCGGTCTGGCAGTAAAAACTATC-3′ and Cre/Esr2 5′-GTGAAACAGCATTGCTGTCACTT-3′; product sizes: Cre positive, 0.1 kb; melting and extension temperature, 60 °C.

### Image and sternum analysis

The lengths of the medio-lateral, dorso-ventral and anterior-posterior axes were measured in E14.5 whole-mount (medio-lateral and anterior-posterior) or E13.5–E14.5 tissue sections (medio-lateral and dorso-ventral) between ribs 2 and 6. In whole mounts, the width was measured at every rib (2–6), and the average width was analysed. The length of the sternum was determined from r2 to r6. In tissue sections, the lengths were measured every 100 μm and averaged for each embryo. Significance was determined using the Students *t*-test. For the analysis of nuclei orientation/cell polarity, whole-mounted sternums were analysed between ribs 3 and 5. Circular analysis was performed using Oriana 4.0; sterna width was measured using AxioVision software by outlining the region of interest. The orientation of nuclei was analysed with CellProfiler2.0. Three-dimensional analysis of confocal *z*-stacks was performed using Imaris and nuclei were reconstructed based on SOX9 immunostaining. Surfaces were built following built-in protocols and the parameters adjusted accordingly. At least three embryos were analysed for each study with the exception of the cell orientation analysis along the dorso-ventral axis where *n*=2 (Supplementary Fig. 3g). The numbers of embryos and cells analysed are stated within the text. Raos spacing test was used to determine the significance of the collective orientation of the nuclei, that is, whether nuclei orientation is random or is polarized. The Mardia–Watson–Wheeler test was used to compare the collective distribution/orientation of nuclei between wild-type and mutant embryos.

Cell proliferation and cell death analysis was carried out in whole-mount sterna or on tissue sections and was analysed using Volocity (PerkinElmer) and ImageJ software.

### Histology and immunolocalisation analyses

Alizarin red and alcian blue skeletal staining to whole mounts and haematoxylin and eosin staining to tissue sections were performed by standard procedures^37^. To orientate the sternum in wax (to cut perpendicular sections throughout the developing sternum), the ventral body wall was marked with carbon particles. For whole-mount antibody staining, E12.5 or older embryos were fixed in 4% paraformaldehyde/PBS for two to four hours at 4 °C. Sternums were dissected, removing nearby skin and muscle, and washed with PBST (PBS, 1% bovine serum albumin (BSA), 0.1% Triton × -100). Sternums were permeabilized in PBST overnight at 4 °C, and then blocked in 5% donkey serum/PBST (PBSTS solution) for 6 h, and then incubated with primary antibodies in PBSTS solution overnight at 4 °C followed by overnight incubation at room temperature. Sternums were washed in PBST (five times, 1 h each), and then incubated with secondary antibodies in PBSTS solution following the same incubation procedure as for primary antibodies. Sternums were washed in PBST (five times, 1 h each), and then washed extensively (two times, 5 h each and then overnight) at room temperature before mounting. After antibody staining, whole sternums were mounted on slides with glass coverslips as spacers to prevent compression of the sternums. Alternatively immunolocalisation was carried out to paraffin wax sections. In brief, sections were dewaxed in xylene, processed through decreasing ethanol solutions into PBS. This was followed by antigen retrieval in citrate buffer (20 mM sodium citrate pH 6) by boiling for 10 min, sections were then washed in PBS, blocked for at least 1 h in 5% serum/PSBT and incubated overnight in the primary antibody at 4 °C. Sections were then washed three times in PBS and incubated in the secondary antibody for at least 1 h at room temperature. This was followed by three more washes in PBS. Sections were mounted in Vectashield hardset. Primary antibodies used include: rabbit anti-Sox9 (Millipore, 1:400 or Santa Cruz 1:50), goat anti-beta-catenin (R&D Systems, 1:100), rat anti-Fat4 and Dchs1 (gift of T. Tanoue^38^, 1:200), mouse anti-phospho-histone H3 (cell signalling, 1:150) and active caspase 3 (cell signalling; 1:150). Secondary antibodies were from Jackson ImmunoResearch, and nuclei were stained with Hoechst 33,342 or 4,6-diamidino-2-phenylindole. Images were obtained using a Leica SP5 confocal.

### Method for sternum culture and live imaging

B6.129(Cg)-Gt(ROSA)26Sortm4(ACTB-tdTomato,-EGFP)Luo/J mice were crossed to FVB.Cg-Tg(CAG-cre/Esr1)5Amc/J mice (Jackson Lab). E10.5 pregnant mice were intraperitonially injected with tamoxifen at dose of 1.2 mg per mouse. The sterna were collected from GFP-positive E12.5 embryos, the skin and most of the rib was removed. Sternums were cultured on Costar Transwell inserts (#3,450) ventral side down in DMEM/F12 (Gibco, Cat. #11,320) with 10% fetal bovine serum plus antibiotics and antimycotics (Gibco, Cat. # 15,240). Live imaging was performed using PerkinElmer spinning disc confocal microscopy at 37 °C and in 5% CO_2_. Images were taken at time intervals of 5 min for up to 10 h. *z*-Stacks (1.5 or 2 μm) were generated using a × 20 objective lens with long working distance (3.5 mm) and water immersion. Both transmitted light and 488 nm laser were used to visualize sternums contour and individual GFP-positive sternum cells, respectively. Live images were analysed with Volocity software.

### *In situ* hybridization

Embryos were fixed in 4% paraformaldehyde at 4 °C overnight, dehydrated into methanol and stored at −20 °C. *In situ* hybridization was carried out using the following probes: Sox9 (ref. 39) and Runx1 (ref. 40). All solutions were RNAse free. Embryos were processed into PBS, treated with 20% H_2_O_2_ in PBS–0.1% Tween (PBST) for 1 h, washed twice in PBST and incubated with 10 μg/ml proteinase K at room temperature (60 min, E13.5 and 45 min for E12.5 embryos), washed in PBST and fixed in 4% paraformaldehyde for 20 min, before incubating in prehybridisation buffer (50% formamide, 5 × SSC, 50 μg ml^−1^ heparin, 50 μg ml^−1^ total yeast RNA, 0.1% SDS) at 70 °C for at least 1 h. Embryos were incubated overnight with the digoxigenin (DIG)-labelled antisense RNA probe (∼1 μg ml^−1^ in prehybridisation buffer). Unbound probe was removed by the following washing steps at 65 °C and 60 °C, respectively: two 30 min washes in 50% formamide, 5 × SSC, 1% SDS followed by two 30 min washes in 50% formamide, 2 × SSC, 0.2% SDS. The embryos were then processed into PBST, blocked in 20% sheep serum in PBST for at least 1 h before incubating in anti-DIG antibody (1/2,000 dilution) overnight at 4 °C. Unbound antibody was removed by at least five washes over 24 h in PBST at room temperature. Bound probe was visualized by staining at pH 9.5 in NMT buffer (100 mM Tris HCl pH 9.5, 50 mM MgCl_2_, 100 mM NaCl) with 5-bromo-4-chloro-3-indolyl-phosphate (BCIP) and nitro blue tetrazolium (NBT) (Boehringer).

## Additional information

**How to cite this article:** Mao, Y. *et al*. Dchs1–Fat4 regulation of polarized cell behaviours during skeletal morphogenesis. *Nat. Commun.* 7:11469 doi: 10.1038/ncomms11469 (2016).

## Supplementary Material

Supplementary Information Supplementary Figures 1-6 and Supplementary Table 1
