# Supplementary material for: Dchs1–Fat4 regulation of polarized cell behaviours during skeletal morphogenesis
Source: Nat Commun. 2016 May 5;7:11469. doi: 10.1038/ncomms11469 (PMC4858749; doi:10.1038/ncomms11469)
Supplement: Supplementary Information — Supplementary Figures 1-6 and Supplementary Table 1 [file ncomms11469-s1.pdf]

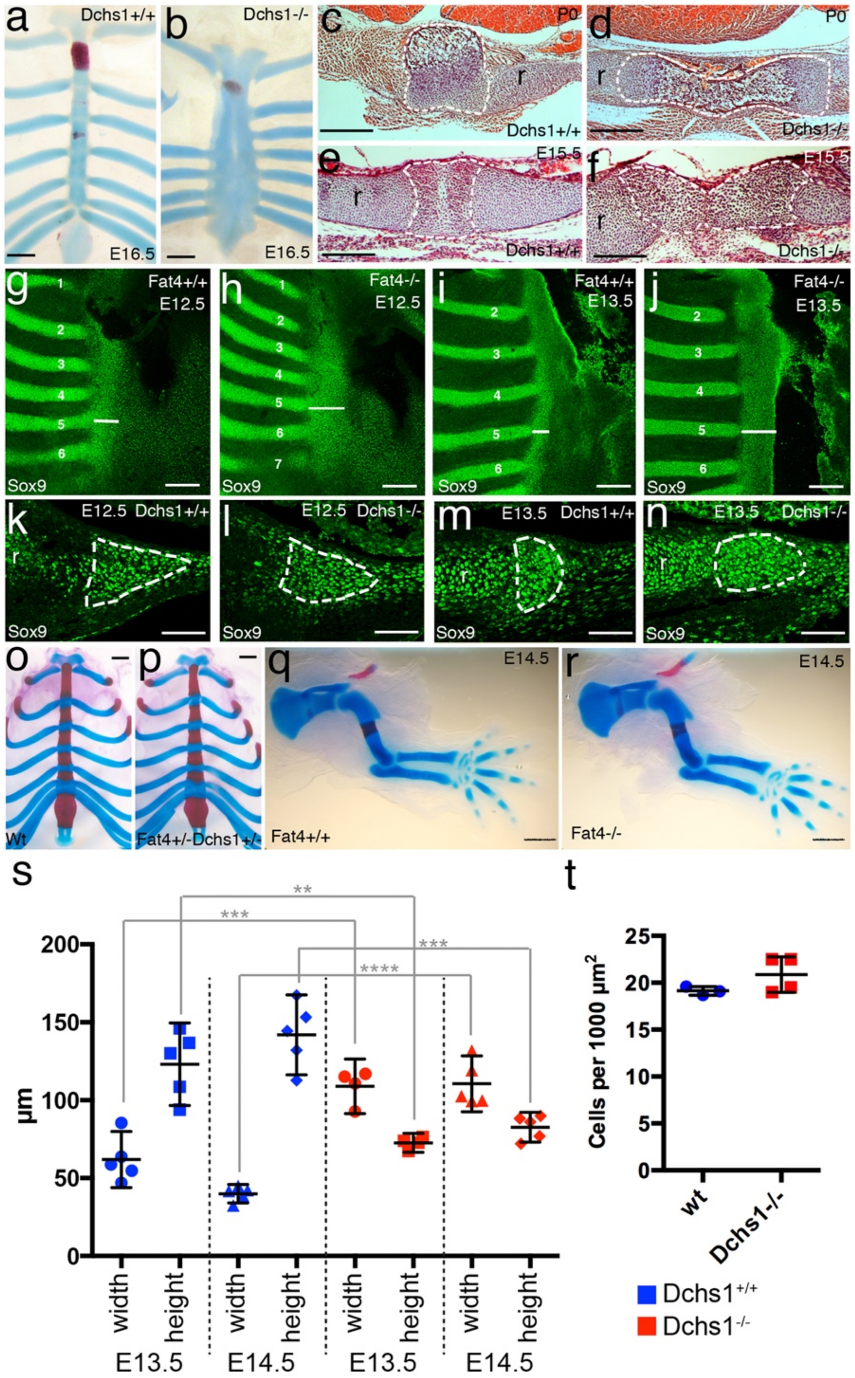

Supplementary Figure 1. Sternum development in *Fat4* and *Dchs1* mutant embryos

Sternal development in wildtype (a, c, e, g, i, k, m, o), $Dchs1^{-/-}$ (b, d, f, l, n), $Fat4^{-/-}$ (h, j) and $Fat4^{+/-}/Dchs1^{+/-}$ (p) mutants. a, b, o, p) Alcian blue (cartilage) and alizarin red (bone) staining of E16.5 (a, b) and P0 (o, p) sterna. c-f) H&E staining of sections through the dorso-ventral axis of P0 (c, d) and E15.5 (e, f) sterna; the sterna are outlined by the white dashes. g-j) flatmounts of E12.5 (g, h) and E13.5 (i, j) sternal bars immunostained for Sox9 (green); the width of the sternal plate is indicated by the white bar and the ribs are numbered. k-n) Sox9 immunostaining (green) of sections through the dorso-ventral axis of developing sternal bars at E12.5 (k, l) and E13.5 (m, n). q, r) Alcian blue and alizarin red staining of E14.5 wildtype (q) and $Fat4^{-/-}$ mutant (r) limbs. Scale bars: (o,p), 1 mM; (a,b), 500 µM (c-f) 200 µM; (k-n), 100 µM. s) Graph showing length in microns across the dorso-ventral and medio-lateral axes of wildtype and $Dchs1^{-/-}$ E13.5 and E14.5 embryos. t) Graph quantifying cell density in E14.5 wildtype and $Dchs1^{-/-}$ embryos. In (s,t) The lines indicate mean (thicker black bar), 25% and 75%. Student's t-test **, $p<0.01$; ***, $p<0.001$; ****, $p<0.0001$.

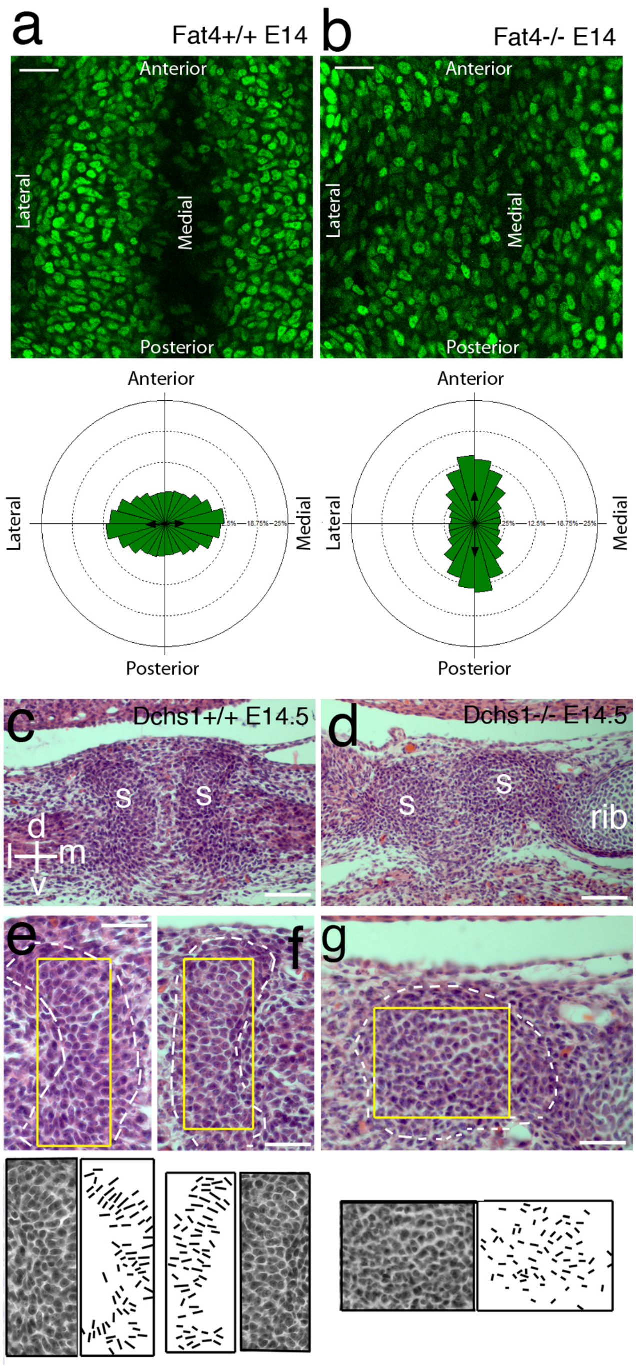

Supplementary Figure 2. The *Fat4*$^{-/-}$ sternal phenotype is not due to a developmental delay.
a,b) flatmounts of E14 wildtype (a) and *Fat4*$^{-/-}$ (b) sternum immunostained for Sox9 (green) to mark sternal nuclei. The anterior-posterior and medial-lateral axes are labelled. Orientation of the nuclei is shown in the Rose Plots below (*Fat4*$^{+/+}$, n=3; *Fat4*$^{-/-}$, n=3). Even after the sternal bands have met in mutant sterna, the sternal cells have not re-orientated across the medio-lateral axis and remain polarised along the anterior-posterior axis. c-g) H&E stained tissue sections through E14.5 wildtype (c, e, f) and *Dchs1*$^{-/-}$ (d, g) sternal bars (s). The dorso-ventral and medio-lateral axes are labeled. (e , f and g) show high power images of the sternal bars shown in (c, d); the sternum is outlined by the white dashed lines. The boxed region is shown in a black and white image below together with a diagram illustrating the orientation of the long axis of the nuclei. The lengths of the individual bars indicate the length of the long axis of the nuclei. Scale bars: (a,b,e, f and g) 25 μm; (c,d), 50 μm.

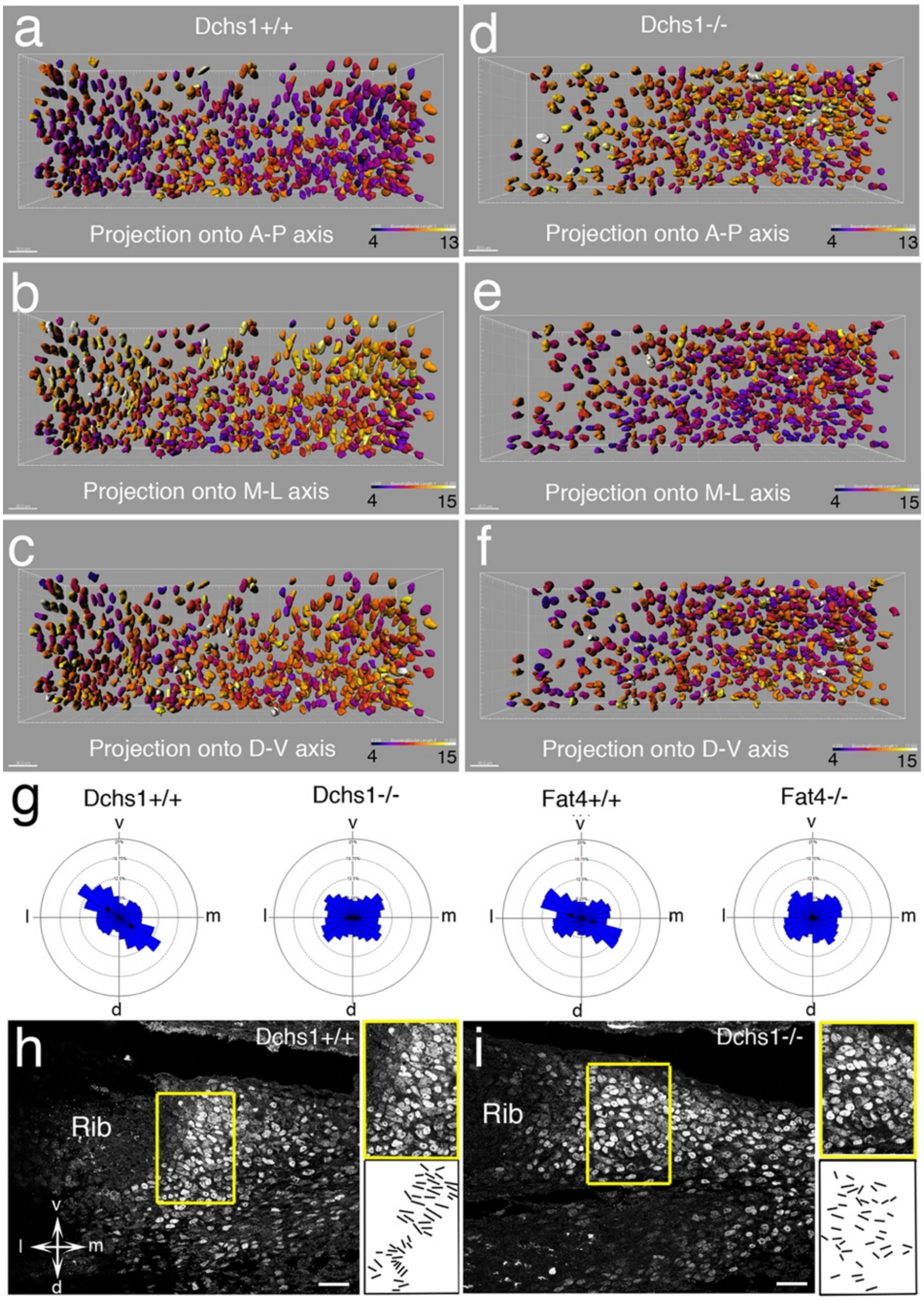

Supplementary Figure 3. 3D analysis of Nuclear orientation

a-f) Examples of analysis of nuclear orientation within 3D confocal stacks of the sternum between ribs 3 to 5 in wildtype and *Dchs1*$^{-/-}$ E13.5 embryos. Volume rendering was performed in Imaris (Bitplane) and the nuclei were reconstructed to show the nuclear length along each axis. The relative length of the nuclei on the A-P, M-L, or D-V axis are indicated by shading from dark purple (short) to yellow and white (long) as indicated in the scale bars. (a,d) show nuclear lengths along anterior-posterior axis, (b,e) nuclear lengths along the medio-lateral axis and (c,f) nuclear lengths along the dorso-ventral axis. Scale bars in μm are indicated on bottom RHS of each image (n=3, >1500 cells analysed per embryo). Quantitation is shown in Fig. 2i. (g) Rose plots of cell orientation along the dorso-ventral axis in E13.5 wildtype, *Dchs1*$^{-/-}$ and *Fat4*$^{-/-}$ embryos (2 *Fat4*$^{-/-}$ and 2 wt littermates; n=2 *Dchs1*$^{-/-}$ plus 2 wt littermates). Between 450-1500 cells analysed per embryo (total number of cells 3690 wt; 1431 *Fat4*$^{-/-}$; 3666 *Dchs1*$^{-/-}$). In these studies orientation has been scored relative to plane of body wall (0°-180° axis). h,i) Sections through the dorso-ventral axis of a E13.5 Dchs1$^{+/+}$ (h) and Dchs1$^{-/-}$ (i) sternum immunostained for the expression of Sox9 (white). Scale bar, 25 μm. The boxed region is shown at higher power on the top RHS together with a diagram below indicating the orientation and length of the long axis of the individual sternal nuclei.

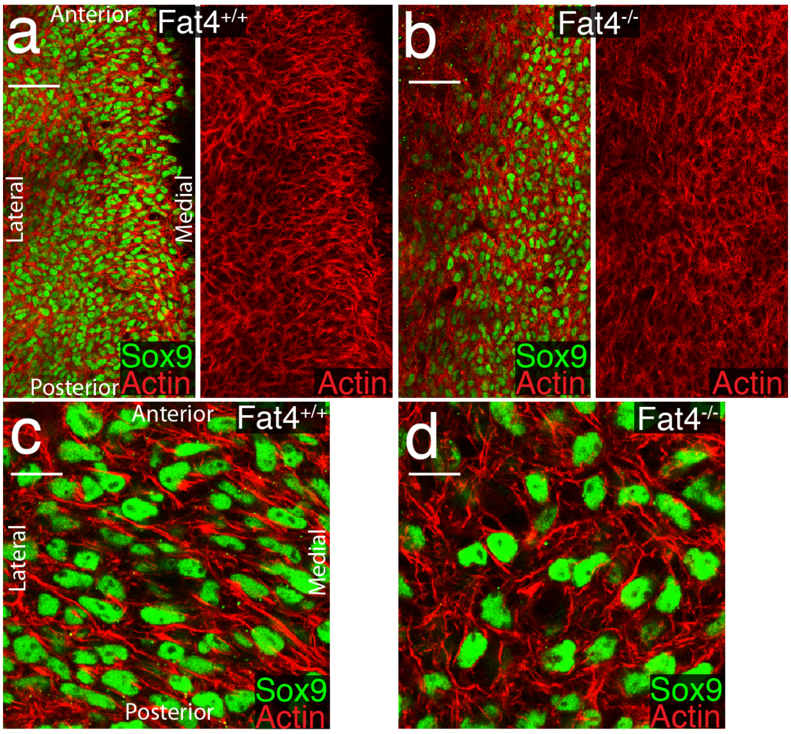

Supplementary Figure 4. F-actin organization in sternal cells

E13.5 sternums from a wild type (a,c) and a *Fat4*$^{-/-}$ embryo (b,d), stained for F-actin (using phalloidin, red), and Sox9 (green). a,b) show lower magnification images, scale bars are 50 μm c,d) show higher magnification images, scale bars are 25 μm.

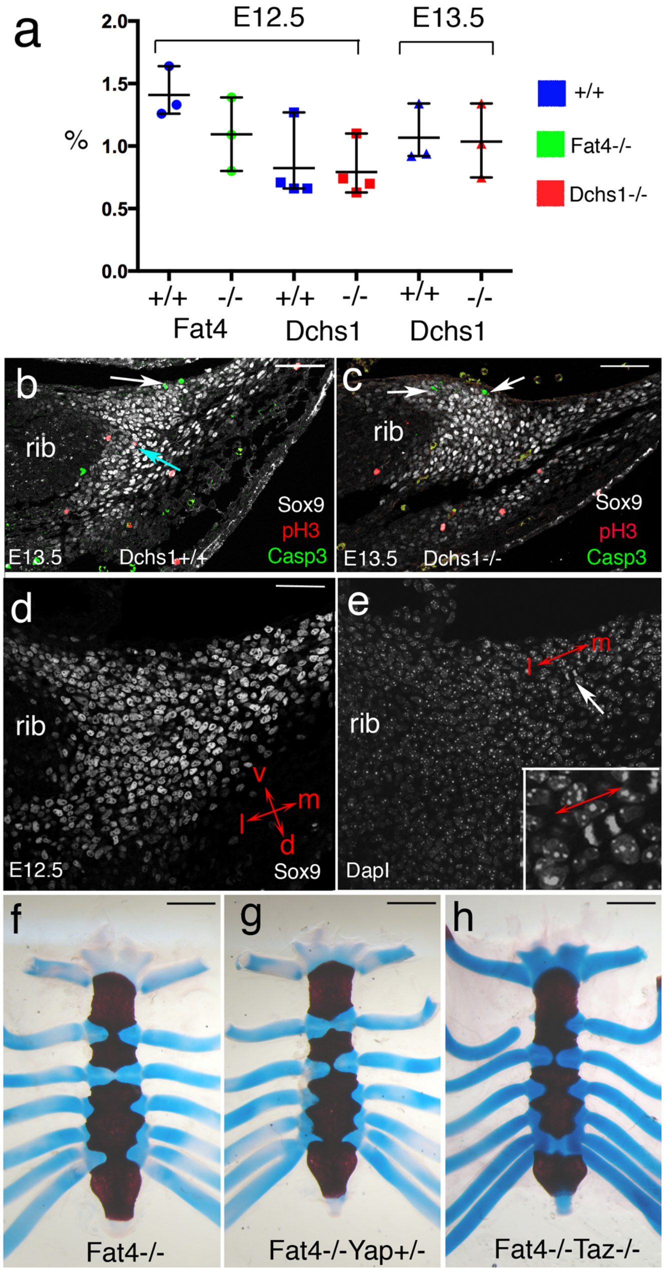

Supplementary Figure 5. Fat4 and Dchs1 do not regulate sternum development through Yap/Taz

a) Rates of mitosis in developing sterna by immunostaining for phospho-histone H3 in E12.5 or E13.5 wildtype/heterozygous versus *Dchs1*$^{-/-}$ or *Fat4*$^{-/-}$ embryos. The lines indicate mean (thicker black bar), 25% and 75%. The total number of cells counted is shown in Supplementary Table 1.

(b, c) show immunolabelling for Sox9 (white), phospho-histone H3 (red) and activated-Caspase3 (green) in a transverse section through *Dchs1*$^{+/+}$ (b) and *Dchs1*$^{-/-}$ (c) E13.5 sterna. Apoptotic cells are indicated by the white arrows; a proliferating cell in (b) is indicated by the blue arrow. d, e) show Sox9 immunostaining (white in d) and DAPI staining (e) of a developing E12.5 Dchs1$^{+/+}$ sternal bar. A cell in late anaphase/early telophase is arrowed in (e) and is also shown in the enlarged box at the right hand bottom corner of the image. The orientation of the divisions was scored relative to the medio-lateral and dorso-ventral axes (indicated by the red lines in (d) and (e). f-h) Alizarin red and alcian blue staining of P0 *Fat4*$^{-/-}$ (f), *Fat4*$^{-/-}$ *Yap*$^{+/-}$ (n=4) (g) and *Fat4*$^{-/-}$*Taz*$^{-/-}$ (n=5) (h) sterna. m-l, medio-lateral; d-v, dorso-ventral, Scale bar, (b,c) 100 µM, (d,e) 50 µM, (f-h), 1 mM

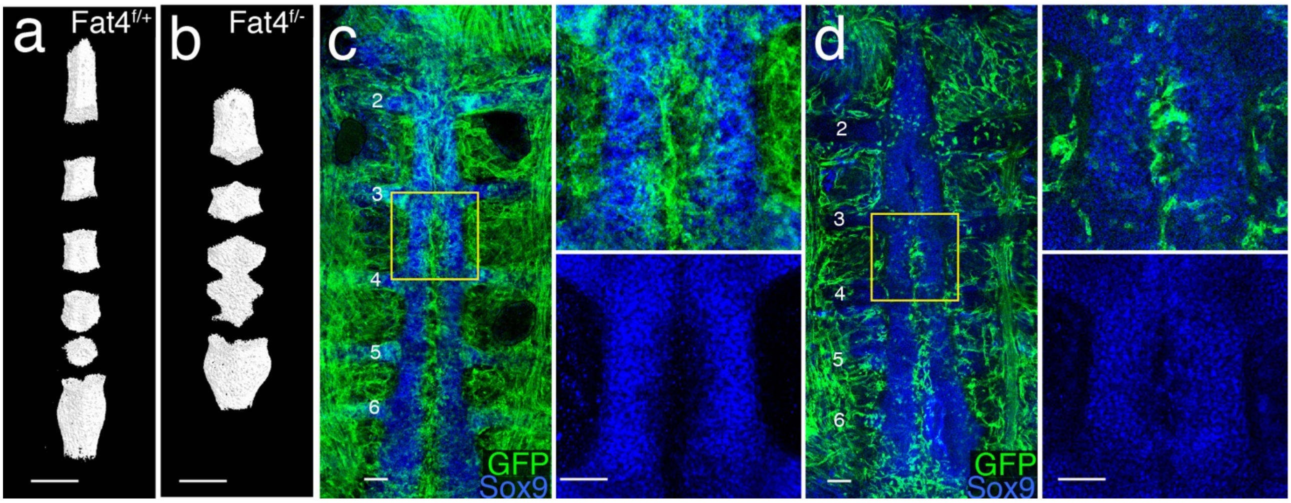

Supplementary Figure 6. Requirement for Fat4 in the sternal mesenchyme

a, b) MicroCT scans of P0 sternums from *Fat4*$^{f/+}$ *Dermo1*^{cre} (control) and *Fat4*$^{f/f}$ *Dermo1*^{cre} (n=3); white areas indicate regions of ossification, anterior is uppermost. c, d) E14.5 sternums with mosaic deletion of *Dchs1* after 2mg of tamoxifen treatment at E10.5; the extent of mosaicism indicated by expression of GFP (green) and sternal nuclei stained for Sox9 (blue). Higher power images of the boxed region shown on the RHS of each image: upper panel shows Sox9 staining and GFP expression. The lower panel only shows Sox9 staining to more clearly show the width of the sternal bars. c) wild-type, d) *Dchs1*$^{f/-}$. Scale bars: (a,b), 1mM (c, d) 100 μM.

Supplementary Table 1 Analysis of cell division orientation in the developing sternum

Genotype and Stage	Number of cells analysed	M-L division	D-V division	45° to M-L axis
E12.5 wildtype	11000 (n=6)	3	0	2
E12.5 *Dchs1*$^{-/-}$	7002 (n=3)	2	1	3
E12.5 *Fat4*$^{-/-}$	6175 (n=3)	2	0	1
E13.5 wildtype	7176 (n=3)	0	0	0
E13.5 *Dchs1*$^{-/-}$	5552 (n=3)	0	0	0

Table showing number of sternal cells analysed and the number of cells in terminal anaphase/early telophase that were dividing along the medio-lateral (M-L), dorso-ventral (D-V) or midway between the M-L/D-V axes. The number of embryos analysed at each stage is shown in brackets.
